# Identification of novel cell glycolysis related gene signature predicting survival in patients with breast cancer

**DOI:** 10.1038/s41598-021-83628-9

**Published:** 2021-02-17

**Authors:** Feng Jiang, Chuyan Wu, Ming Wang, Ke Wei, Jimei Wang

**Affiliations:** 1 Department of Neonatology, Obstetrics and Gynecology Hospital of Fudan University, No. 419, Fangxie Road, Shanghai, 200011 China; 2 Department of Rehabilitation Medicine, The First Affiliated Hospital of Nanjing Medical University, Nanjing, 210029 China; 3 Plastic Surgery Department, The First Affiliated Hospital of Nanjing Medical University, Nanjing, 210029 China; 4 Medical Department, The First Affiliated Hospital of Nanjing Medical University, Nanjing, 210029 China

**Keywords:** Computational biology and bioinformatics, Genetics

## Abstract

One of the most frequently identified tumors and a contributing cause of death in women is breast cancer (BC). Many biomarkers associated with survival and prognosis were identified in previous studies through database mining. Nevertheless, the predictive capabilities of single-gene biomarkers are not accurate enough. Genetic signatures can be an enhanced prediction method. This research analyzed data from The Cancer Genome Atlas (TCGA) for the detection of a new genetic signature to predict BC prognosis. Profiling of mRNA expression was carried out in samples of patients with TCGA BC (n = 1222). Gene set enrichment research has been undertaken to classify gene sets that vary greatly between BC tissues and normal tissues. Cox models for additive hazards regression were used to classify genes that were strongly linked to overall survival. A subsequent Cox regression multivariate analysis was used to construct a predictive risk parameter model. Kaplan–Meier survival predictions and log-rank validation have been used to verify the value of risk prediction parameters. Seven genes (PGK1, CACNA1H, IL13RA1, SDC1, AK3, NUP43, SDC3) correlated with glycolysis were shown to be strongly linked to overall survival. Depending on the 7-gene-signature, 1222 BC patients were classified into subgroups of high/low-risk. Certain variables have not impaired the prognostic potential of the seven-gene signature. A seven-gene signature correlated with cellular glycolysis was developed to predict the survival of BC patients. The results include insight into cellular glycolysis mechanisms and the detection of patients with poor BC prognosis.

Breast cancer is the world's most prevalent form of cancer with high morbidity^1,2^. According to Global Cancer Statistics 2018, there will be nearly 2.1 million new cases diagnosed globally and approximately 62,000 deaths. Patients with the same development will therefore have varying prognoses and treatment reactions^3,4^. Efficient BC biomarkers must also be discovered to assess prognoses and classify potential patients with elevated BC risk.

Many biomarkers for BC, such as ER and HER2, have been established^5,6^. Scientists have built various patient genome repositories through developments in high-performance sequencing to help them recognize genetic shifts more systematically^7,8^. Through database mining, we have identified thousands of biomarkers that could interact with the prognosis of tumor patients. Consequently, the predictive power of single-gene biomarkers is also inadequate. Work has also shown that determining the genetic properties of many genes will improve prediction^9,10^. More precise treatment approaches may be driven by multigenic prognostic characteristics from primary tumor biopsy. The latest studies also analyzed the role of multiple-gene signatures on BC for prognosis evaluation and for the detection of potentially high BC risk patients^11,12^.

In this research, genes were identified by doing gene set enrichment analysis (GSEA). In order to classify biomarkers, differential analytics usually include analyzing variations in expression between groups with a focus on genes with substantially controlled rates of expression^13^. However, this approach can effectively remove genes that do not display significant variations in expression, which do provide essential biological knowledge or demonstrate biological value. In order to check cumulative expression of multiple genes, GSEA as an upcoming computational tool will not involve a specific distinct gene threshold or comprehensive expertise. This shows general data patterns. This method thus strengthens the comparative study of biological expression and biological relevance^14^.

Accordingly, in this research, we have been analyzing details from the Cancer Genome Atlas (TCGA) in order to establish a specific genetic signature for BC forecasting. In order to map the marker genome of 1222 patients with BC, we used mRNA expression results from TCGA. In order to accurately predict patient results, we find 251 mRNAs that are substantially linked to glycolysis and established a seven-gene risk profile. Interestingly, glycolysis risk factors may be used to accurately determine the prognosis of high-risk patients. The gene signature linked to a novel cell glycolysis was identified and validated.

## Methods

### Patient clinical and mRNA expression data collection

We collected clinical evidence and profiles of mRNA expression for TCGA (https://cancergenome.nih.gov/) breast cancer patients. The research included clinical details from 1222 patients and the age, sex, step, T-classification, N-classification, M-classification (Table 1).

**Table 1:** Gene sets enriched in Breast cancer.

GS follow link to MSigDB	Size	ES	NOM *p* value	Rank at MAX
Medullary ductal	202	0.56	0.035	7425
ESR1 targets not via AKT1	211	0.59	< 0.001	7548
ESR1 targets via AKT1	279	0.57	0.002	6697
G2M arrest	117	0.47	0.01	4359
BC basal	123	0.52	0.011	5593
BC luminal basal	379	0.71	< 0.001	8605
BC luminal mesenchymal	447	0.73	< 0.001	7859
HA glycolysis	199	0.58	< 0.001	5817
RE glycolysis	71	0.64	< 0.001	6296

### Gene set enrichment analysis

We carried out GSEA (http://www.broadinstitute.org/gsea/index.jsp) to decide if the gene sets found varied greatly between the BC and normal groups. Then the expression levels of 56,753 mRNAs in BC and neighboring noncancerous tissues were examined. Ultimately, we marked the functions for further study with normalized *p* values (*p* < 0.05).

### Data analysis and estimation of risk parameters

RNA expression were downloaded from the TCGA data portal. Univariate Cox regression study was used to classify genes that were then exposed to multivariate Cox regression to validate prognostic genes and obtain the coefficient. The identified mRNAs were subsequently divided into risky form and protective (0 < HR < 1) sort (hazards ratio, HR > 1). We built a risk-parameter function as follows by a linear combination of expression values of filtered genes weighted by their coefficients: Risk Parameter = (βn * expression of gene n). The 1222 patients were classified by the median risk criterion into high-risk and low-risk subgroups.

### Statistical analysis

We used the survival curves of Kaplan–Meier and the log-rank test to approximate the significance of the risk parameter. We performed multivariate analyzes of the Cox and data stratification to check if age, stage, T-classification, N-classification, or M-classification of the risk parameter is independent of clinical features used as covariates. Statistically significant was a *p* < 0.05. Statistical research was carried out using the program SPSS 19.0 (SPSS, Inc., Chicago, IL, USA).

### Consent for publication

All listed authors took part actively in the research and read and approved the manuscript submitted.

## Results

### Primary GSEA gene screening

**Figure 1:**
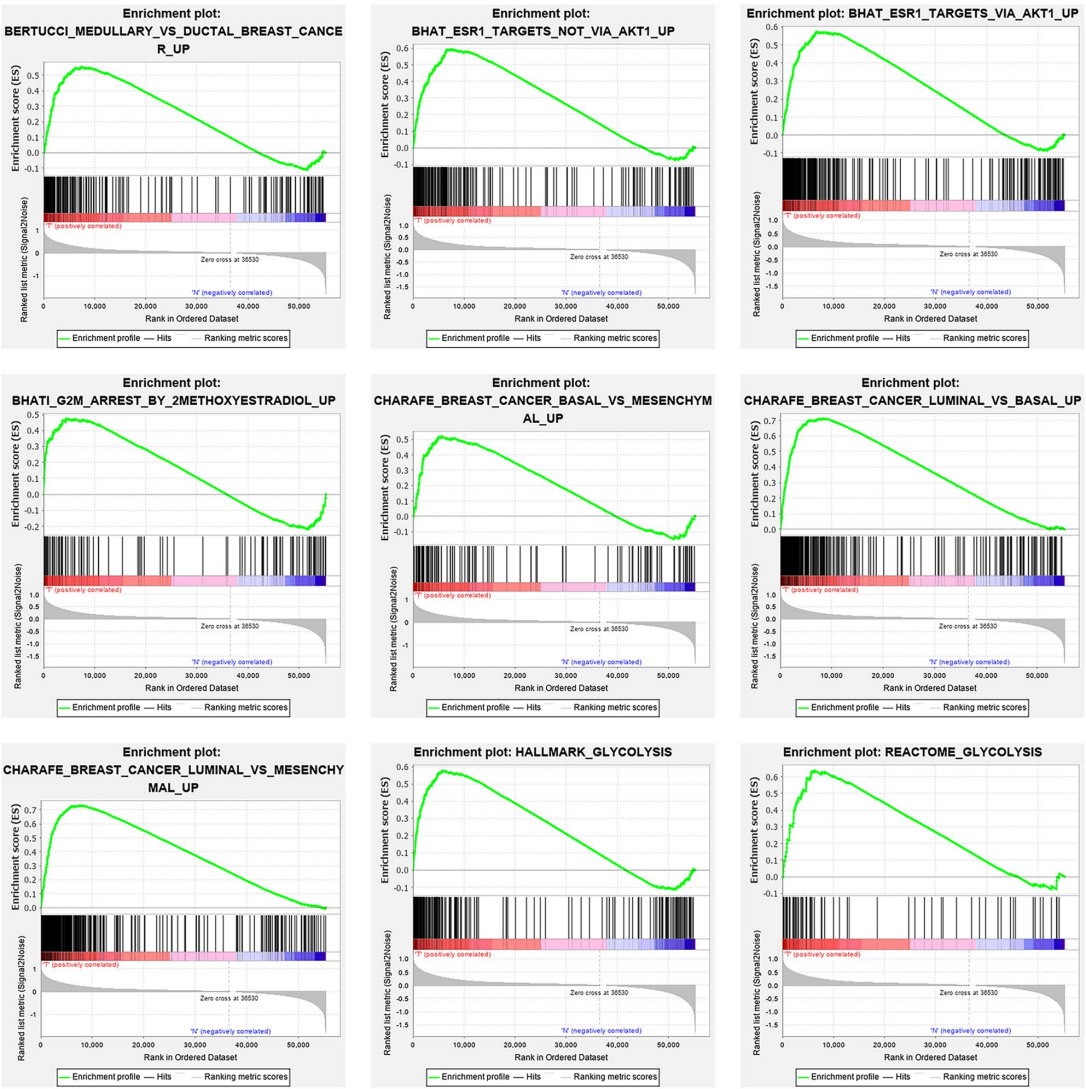
enrichment of nine gene sets with major variations between BC tissues and noncancerous tissues by GSEA.

We collected clinical features of 1222 BC patients along with expression details from a TCGA sample of 56,753 mRNAs. We conducted GSEA to decide if the genes found varied substantially between BC tissues and normal tissues. We validated 25 gene sets that were upregulated in BC. 9 gene sets, Medullary ductal, ESR1 targets not via AKT1, ESR2 targets via AKT1, G2M arrest, BC basal, BC luminal basal, BC luminal mesenchymal, HA glycolysis, RE glycolysis were significantly enriched (Table 1; Fig. 1). We then filtered the top-ranking function, glycolysis (*p* < 0.001), among 276 genes for further study.

### Identification of survival linked mRNAs related to glycolysis

First, the univariate Cox regression study of 276 genes was performed for preliminary screening, and 251 genes were generated (*p* < 0.05). Then, a multivariate Cox Regression analysis was conducted to further analyze the relationship between the 251 mRNA expression and patient survival profiles and to classify the most important mRNA combinations using the stepwise elimination process. 251 mRNAs were verified and 7 ((PGK1, CACNA1H, IL13RA1, SDC1, AK3, NUP43, SDC3)) of the 251 mRNAs were validated as independent BC prediction markers. The filtering mRNA is divided into risky type (PGK1, CACNA1H, IL13RA1, SDC1, NUP43), with poorer survival associated HR > 1 as well as the protective type (AK3, SDC3) with better survival associated HR < 1 (Table 2).

**Table 2:** The detailed information of seven prognostic mRNAs significantly associated with overall survival in patients with breast cancer.

mRNA	Ensemble ID	Location	B(Cox)	HR	*p*
PGK1	ENSG00000102144	chr X: 78104248–78129295	0.006297	1.006317	< 0.0001
CACNA1H	ENSG00000196557	chr 16: 1153106–1221772	0.013659	1.013753	0.0154
IL13RA1	ENSG00000131724	chr X: 118726954–118794533	0.006425	1.006446	0.0102
SDC1	ENSG00000115884	chr 2: 20200797–20225433	0.002093	1.002095	0.0024
AK3	ENSG00000147853	chr 9: 4709556–4742043	− 0.02549	0.974832	0.0422
NUP43	ENSG00000120253	chr 6: 149724315–149746529	0.043829	1.044804	0.0011
SDC3	ENSG00000162512	chr 1: 30869466–30909735	− 0.0225	0.977754	0.0247

**Figure 2:**
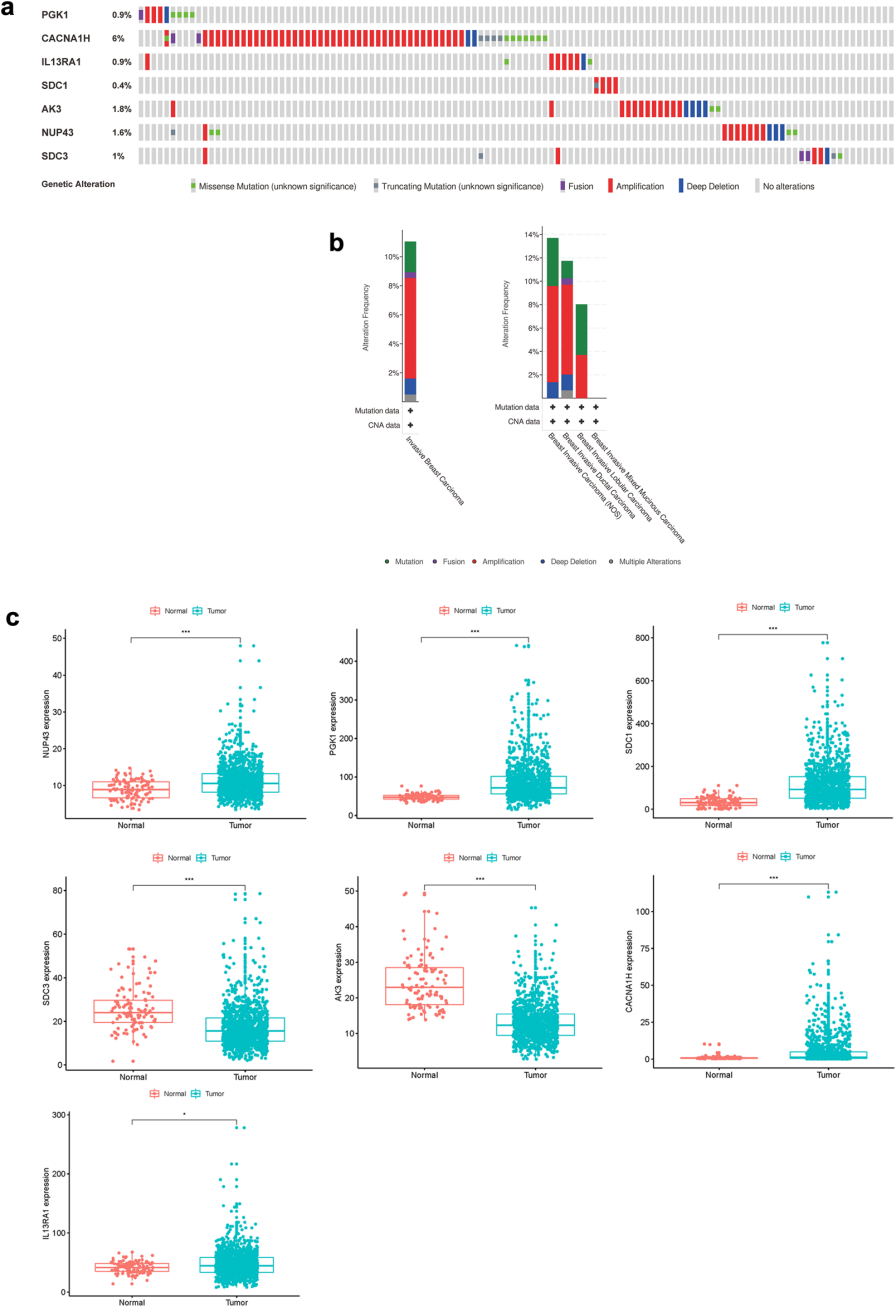
Identification of patient survival mRNAs. (**a**) Alteration of the selected genes in clinical samples. (**b**) Modification of chosen genes in various pathological forms of BC. (**c**) Multiple expression of seven genes selected.

The differences in 7 filtered genes were then analyzed with the study of 996 BC samples from cBioPortal (http://cbioportal.org). The findings found that 110 (11.04%) of the sequenced cases changed the queried genes. The PGK1 gene included 3 amplification samples, 1 deep deletion samples, 4 mutation samples, and 1 sample with fusion. The CACNA1H gene was altered in 5.72% of cases, showing various changes. The IL13RA1 gene was altered in 0.9% of cases. The SDC1 gene was altered in 0.4% of cases. The AK3 gene was altered in 1.81% of cases, and the NUP43 and SDC3 genes were altered in 1.61% and 1% of cases, respectively (Fig. 2a).

Relevant variations in the selected genes is important in some forms of cancer. 4.11% of variations in invasive breast carcinoma (NOS) were mutations, 8.22% were amplifications, and 1.37% were deep deletions. In breast invasive ductal carcinoma, 1.48% of changes were mutations, 0.54% were fusions, 7.69% were amplifications, 1.35% were deep deletions and 0.67% were multiple alterations. In breast invasive lobular carcinoma, Mutation was the most eminent alteration (Fig. 2b).

The expression variations of seven genes were also related across adjacent normal tissues (n = 113) (wilcoxon test was used to test the differential gene expression). We find that the expression rates of the 7 genes in BC tissues were substantially enhanced or decreased (Fig. 2c).

### Creating a seven-mRNA signature to forecast patient results

**Figure 3:**
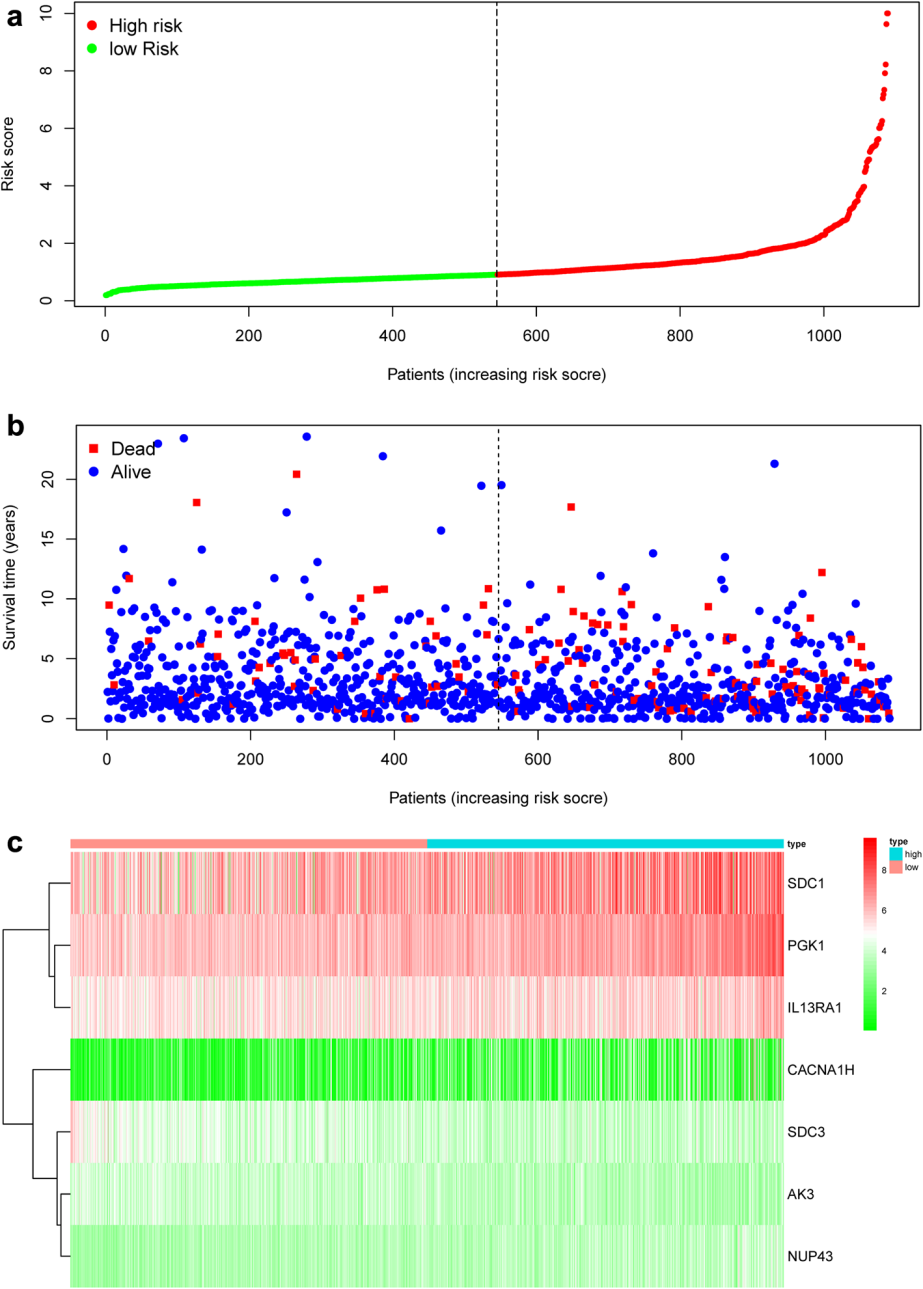
The risk parameter-associated seven-mRNA signature predicts OS in patients with breast cancer. (**a**) The distribution of risk parameter of mRNA in each patient. (**b**) Survival days of BC patients with increasing risk parameters. (**c**) A heatmap of the expression profile of seven genes. Red indicates upregulated genes and light green indicates downregulated genes.

**Figure 4:**
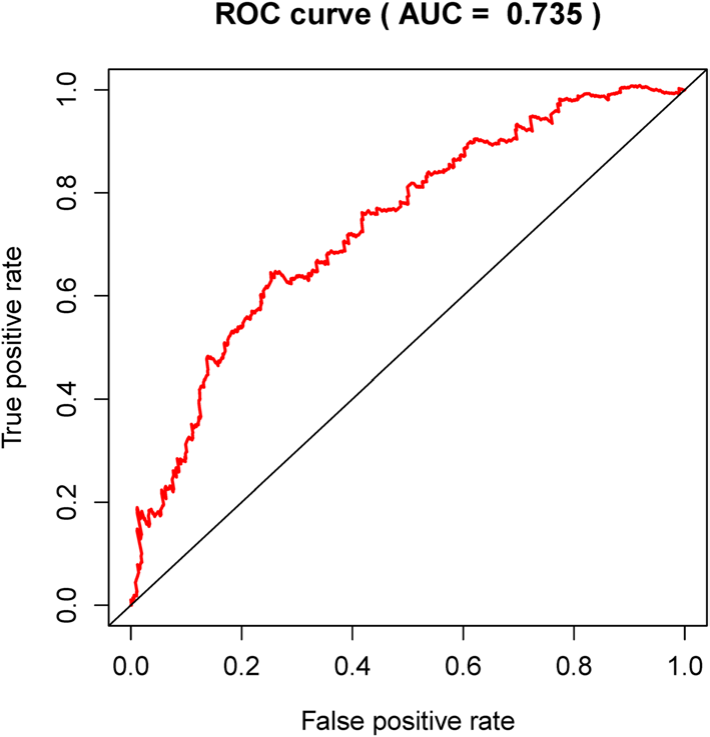
Time-dependence ROC curve according to the 5-year survival of the area under the AUC value.

We have developed the following prognostic risk-parameter formula by linearly combining the expression values of selected genes weighed by their coefficients from the multivariate Cox regression analysis. Risk parameter = 0.8585 * expression of PGK1 + 2.4005 * expression of CACNA1H + 0.1947 * expression of IL13RA1 + 1.8067 * expression of SDC1 + 0.3409 * expression of NUP43 − 0.8953 * expression of AK3 − 0.5676 * expression of SDC3. We calculated parameters and assigned one risk parameter to each patient. We measured parameters and allocated each patient one risk parameter. We then separated patients into high-risk and low-risk subgroups with the median in an upwards order (Fig. 3a). In estimating survival in BC cases, Time-dependent ROC curve analysis according to the 5-year survival of the area under the AUC value was 0.735 (Fig. 4), showing good prognostic performance in predicting survival. Each patient's survival time as shown in Fig. 3b. The high-risk parameter participants reported fewer survival, while the low-risk parameter cases recorded fewer mortality. In comparison, a heat map shows 7 mRNAs expression profiles (Fig. 3c). Compared to the low-risk group, the expression level of risky-type mRNA (PGK1, CACNA1H, IL13RA1, SDC1, NUP43) was higher in the high-risk group. In contrast, the expression level of high-risk group (AK3, SDC3) was lower than that in the low-risk group.

### Seven-mRNA signature risk parameter is an independent prognostic predictor

**Figure 5:**
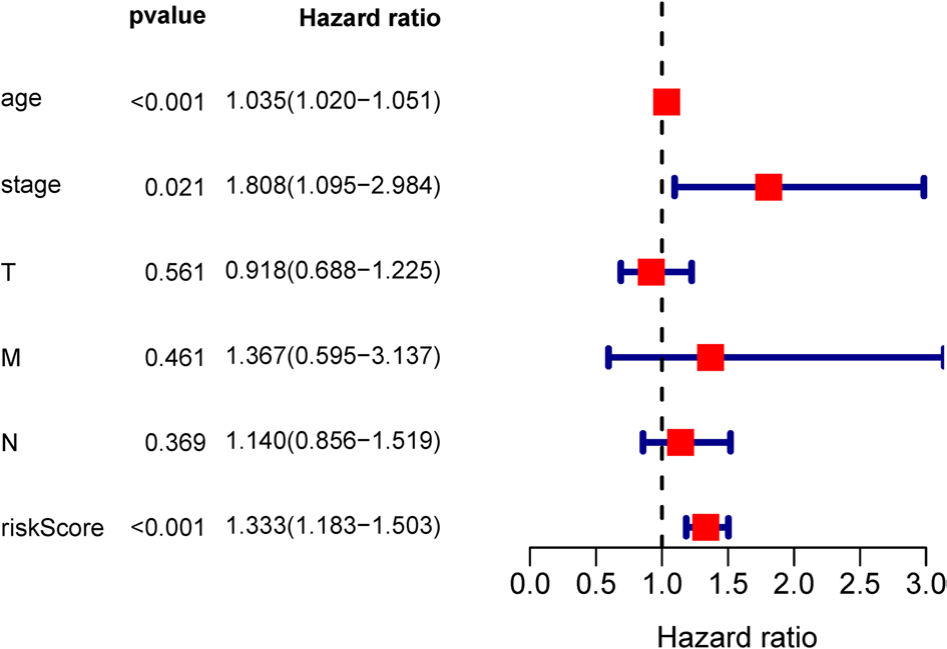
Forest plot of multivariate COX regression analysis.

Through univariate and multivariate regression we were contrasting the prognostic significance of the risk factors with the factors in clinical pathology (Table 3). Samples have been chosen with well established clinical data. Among the 914 patients, 98.8% were female. Among the 914 patients, 75.9% had stage I–II disease, and the remaining 24.1% patients had stage III–IV disease. Among 914 patients, 85.1% patients had I–II T classification, 49.3% had N0 classification and 98.1% had M0 classification. Based on the data given above, we have defined risk parameters, age, stage, T-classification, N-classification and M-classification as independent prediction indicators, as these variables indicated significant differences in univariate analysis and age, the stage showed significant differences in multivariable analysis (Table 4; Fig. 5). In fact, there were important prognostic values of *p* < 0.05 (HR = 1.333) in risk parameters.

**Table 3:** Clinical pathological parameters of patients with Breast cancer in this study.

Clinical pathological parameters	N	%	Dead number
**Age**			
≥ 66	243	26.6	193
< 66	671	73.4	597
**Gender**			
Female	903	98.8	780
Male	11	1.2	10
**Stage**			
I–II	694	75.9	622
III–IV	220	24.1	168
**T classification**			
I–II	778	85.1	688
III–IV	136	14.9	102
**N classification**			
N0	451	49.3	410
N1–3	463	50.7	380
**M classification**			
M0	897	98.1	786
M1	17	1.9	4

**Table 4:** Univariable and multivariable analyses for each clinical feature.

Clinical feature	Number	Univariate analysis			Multivariate analysis		
		HR	95%CI of HR	*p* value	HR	95%CI of HR	*p* value
Risk parameter (high-risk/low-risk)	544/545	1.395	1.253–1.553	< 0.001	1.333	1.183–1.503	< 0.001
Age (≥ 66/< 66)	319/769	1.035	1.02–1.05	< 0.001	1.035	1.02–1.051	< 0.001
Stage (I–II/III–IV)	799/266	2.166	1.713–2.738	< 0.001	1.808	1.095–2.984	0.021
T (I–II/III–IV)	909/176	1.544	1.245–1.915	< 0.001	0.918	0.688–1.225	0.561
N (0/1–3)	512/556	1.7	1.412–2.047	< 0.001	1.14	1.183–1.503	0.369
M (0/1)	900/22	6.419	3.6–11.446	< 0.001	1.367	0.595–3.137	0.461

### Verification of seven-mRNA signature by K-M survival predictions for prognosis

**Figure 6:**
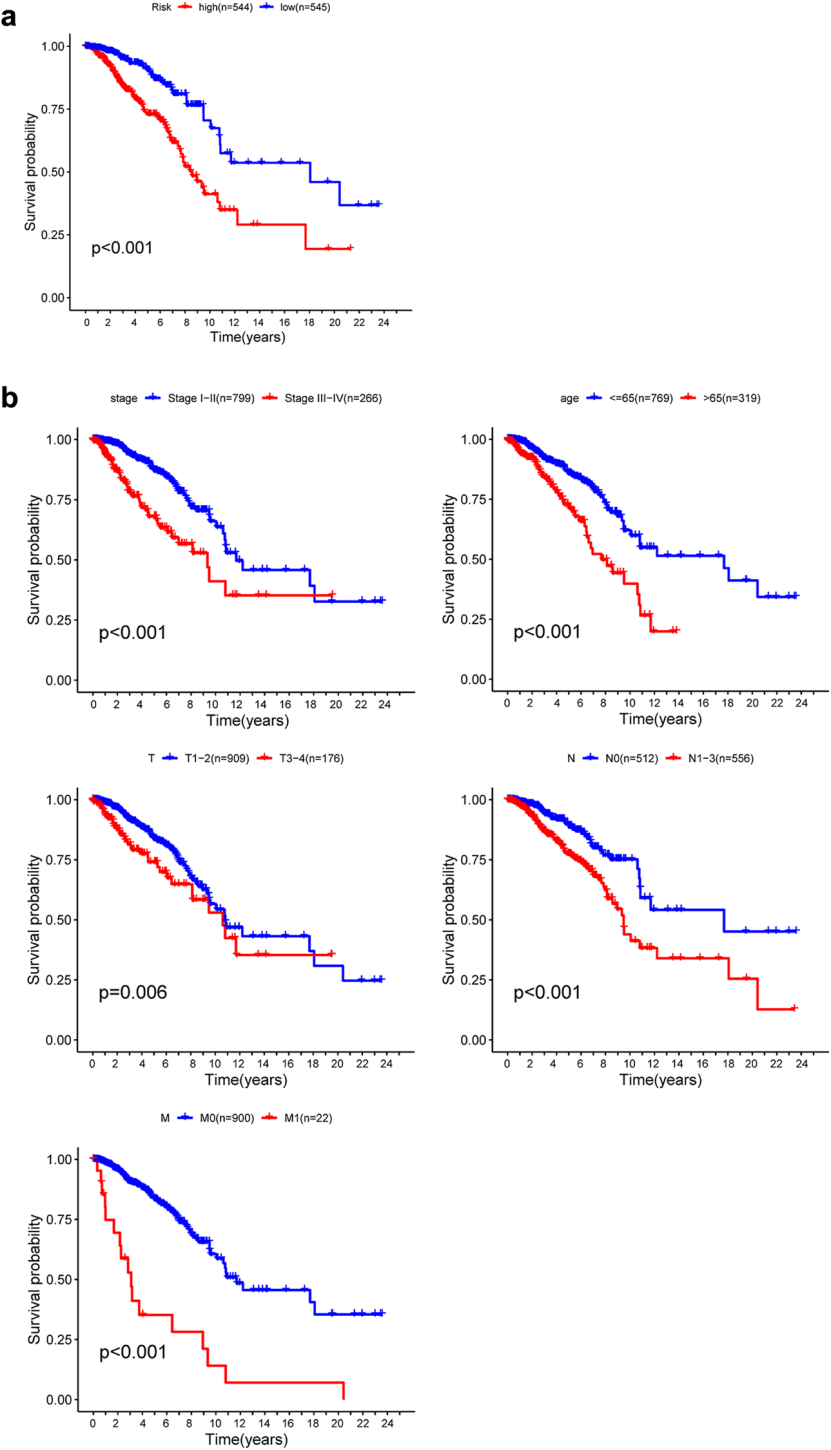
Kaplan–Meier survival study in TCGA data set for BC patients. (**a**) K–M survival curve for high/low risk BC patients. (**b**) Age, Stage, T-classification, N-classification and M-classification features involve patients survival in clinical features.

K-M survival estimates and a log-rank study showed a poor prognosis for patients in the high-risk group (Fig. 6a). Univariate Cox OS regression analysis reported several clinicopathologic parameters that predict BC survival, such as age, stage, T classification, N classification and M classification. In order to validate the above conclusions, we then used Kaplan–Meier survival estimates, which gave clear findings. Patients older than 66 years with disease stage III–IV were associated with poor prognosis (Fig. 6b). These results further confirmed the reliability of the analysis.

**Figure 7:**
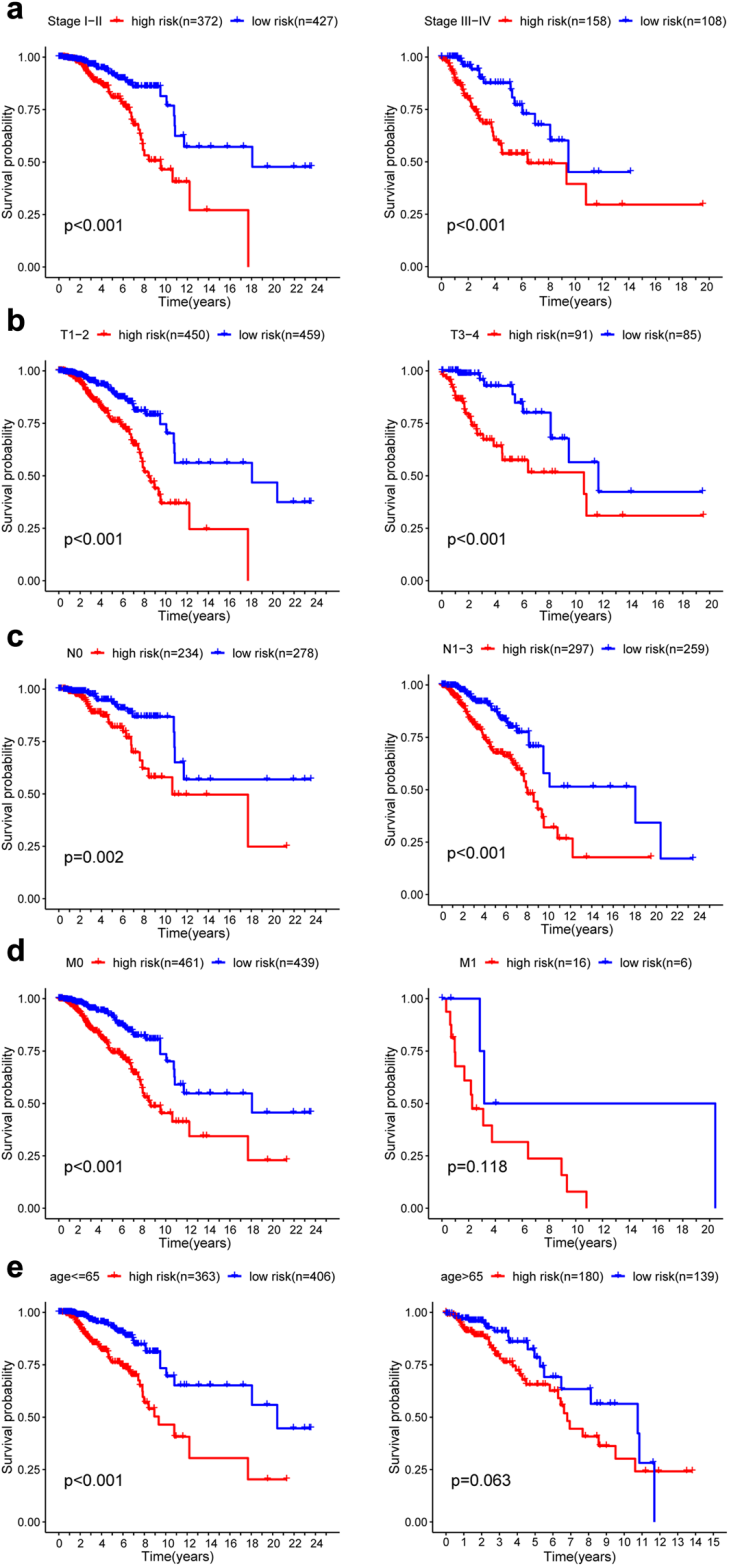
Kaplan–Meier curves for the prognostic value of the signature of risk parameter in each clinical feature for the patients. (**a**) Age, (**b**) stage, (**c**) T classification, (**d**) N classification, (**e**) M classification.

Further stratified analysis was performed for data mining. As shown from the K–M curve, regardless of stage, T classification, N classification and M classification, the 7-mRNA signature was a stable prognostic marker for breast cancer patients who were in the high-risk group and had a poor prognosis (Fig. 7a–c). However, When patients with BC are divided into two subgroups by age (> 65 or ≤ 65 years) and M classification, however, the risk parameter could no longer be used separately as the prognostic predictor for the age of ≤ 66 years (Fig. 7e) subgroup and M1 subgroup (Fig. 7d). We also download GSE25066 datasets from the GEO database, for each gene included in our research, we divided the dataset into high level group and low level group, we observed that high level of AK3, CACNA1H, IL13RA1, SDC3 had better prognosis, but low level of PGK1 and SDC1 had poor prognosis. There was no significant difference between high and low lever group of NUP43 (Fig. S1). More analysis is needed here.

## Discussion

The latest findings have found that clinical anatomy, including age and metastatic diagnosis, is not adequate to accurately determine the outcome of cancer patients^15^. An increasing number of mRNAs were shown to be tumor development biomarkers or prognosis, and the therapeutic significance of the biomarkers was assessed^16,17^. For e.g., Shao et al. confirmed that low expression of DKK2 is an independent prognostic biomarker of shorter progression-free survival in breast cancer patients^18^. Cox multivariate study of the proportional frequency regression model was often used to check that elevated tumor protein HPSE expression patients had improved outcomes, and this protein was also deemed a prognostic predictor for gastric cancer patients^19^. Nevertheless, these biomarkers were also not adequate to assess patient prognoses independently^20^. In particular, several variables may influence the rates of single gene expression that preclude the use of such measures as accurate and independent prognostic measures. A mathematical model consisting of genetic markers for several associated genes was therefore used in tandem with the predictive effects of each variable gene in order to enhance prediction^21^. When determining the prognosis of tumor patients, the model is significantly more reliable than utilizing standard biomarkers, which results when extensive usage of the model.

The accelerated advancement of high-performance genetic sequencing technologies established the foundation for large-scale biological data analysis. Huge amounts of genomics were collected to classify novel diagnostic, prognostic and pharmacological biomarkers by human specimens. A modern prognostic signature has been developed in recent research utilizing microarray and RNA sequence evidence for rates of gene expression or mutations. For detection and testing, a Cox proportional hazards regression model was used. In the current analysis, 9 roles with substantial variations in GSEA were established. As mentioned above, we have chosen the top-ranking feature to filter genes relating to patient survival prediction instead of large-scale discovery. The study of Cox Regression Univariate and Multivariate was conducted to assess the prognostic significance of the seven gene combination for BC patients. This selected risk profile can be more specific and powerful for predicting positive clinical results and can be a tool for classifying BC patients than other known prognostic evaluation markers.

In this research, bioinformatics approaches were used to examine the features and clinical significance of the mRNA risk factors and to test a new way of identifying possible prognostic markers. This work complements BC's earlier interpretation and offers a framework for potential BC studies. In TCGA, we used the BC data collection to gather genes linked to glycolysis and to compare the standard and BC tissue results. Kaplan–Meier survival estimates revealed that patients with low-risk parameters had a better prognosis. For BC cases, risk parameter identification and estimation have important clinical consequences. Nevertheless, we could only use OS to determine patient prognosis due to lack of patient metastasis and recurrence details in the TCGA database, which is one drawback of our work. In addition, the risk parameter may forecast the prognosis of BC patients in all subgroups, except in subgroups of < 66 years of age, in stratified studies. There is no obvious explanation why this disparity needs further analysis.

Uncontrolled cell proliferation characterizes the tumor and not only lacks cell cycle regulation, but facilitates the metabolism of cell-energy and eventually contributes to tumor cell growth and differentiation. Cellular energy is extracted primarily from the oxidation of sugar, and ATP provides much electricity. In the 1920s, the German scientist Otto Warburg noticed defects in hepatoma cell energy metabolism^22^. When oxygen is available, tumor cells mainly depend on metabolism for glycolysis and use vast amounts of glucose followed by the development of lactic acid^23,24^. This condition was called an aerobic glycolysis or a Warburg effect of irregular glucose metabolism. Studies have shown that tumor cells may control ATP synthesis precisely by controlling the uptake of substrate and glycolysis-related enzymes to allow them to respond rapidly to the nutrient microenvironment, fulfill the energy and nutrients requirements for malignant proliferation^25^. Therefore, cancer metabolism, which is directly linked to the Warburg effect, plays a significant role in the conservation of the relationship between the oxygen sensor and the signal system of the nutrient sensor^26^. This indicates that aerobic glycolysis requires a complex action system. The proliferation of tumor cells continues at a pace beyond cellular capacity, and thus excessive cell intake of oxygen or nutrients will contribute to a hypoxic and low-sugar and acidic tumor micro-environment which is more prominent in large tumors. While not all tumors have the Warburg effect, cellular energy defects are well-known to be a characteristic of tumor cells^27^. The Warburg influence has emerged in multiple malignant cancers, such as lung cancer, prostate cancer, pancreatic cancer and colon cancer, following more than 90 years of continuing study and testing. Recent findings have shown that aerobic glycolysis plays a significant function in the growth of BC^28^. Metabolism in BC cells showed a higher glycolysis rate and a lower glucose oxidation rate. The GLUT6 transportation and glycolytic-lipogenic metabolism will depend on tumor cells in order to function. Highly segregated BC showed substantially fewer expression of GLUT1 and GLUT3 than poorly segregated tumors^29^. Several experiments also estimated the longevity of BC patients utilizing cellular glycolysis-associated genes. MAP3K1 elimination, for example, essentially stops the growth and creation of BC^30^. The presentation of HPSE is a closely correlated independent prognostic predictor of weak prognosis in BC. However, gene markers associated with glycolysis have not been established to predict BC prognosis. Using bioinformatics techniques, we calculated and demonstrated its prognostic significance in BC for the genetic characteristics linked to cellular glycolysis (PGK1, CACNA1H, IL13RA1, SDC1, AK3, NUP43, SDC3). The PGK1 gene provides suggestions for the formation of an enzyme kinase phosphoglycerate. This enzyme is present in the human body in cells and tissues, and is involved in a vital energy processing mechanism called glycolysis. Papandreou et al. demonstrated hypoxic adaptation, which reduced mitochondrial oxygen intake by downstream HIF activation of PDK1 in addition to an improved production of glycolytic enzymes^31^. CACNA1H modulates Ca^2+^ levels and the synaptic vesicle cycle but the mechanism related to glycolysis is still unknown. Other genes IL13RA1, SDC1, AK3, NUP43, SDC3 were all enriched in glycolysis, but the mechanisms need to be further investigated.

## Conclusion

We established a seven-gene risk profile linked to cell glycolysis that predicts the prognosis for BC patients with an elevated risk parameter that suggests a poorer statement. In clinical practice, the signature can be used as a tool. Such studies give insight into the processes of cellular glycolysis and classify poor BC prognosis patients..

## Supplementary Information

Supplementary Figure S1.

Supplementary Information 1.

Supplementary Information 2.

Supplementary Legend.

## Data Availability

The generated and analyzed datasets of the current research are available in TCGA (http://cancergenome.nih.gov/abouttcga) and cBioPortal.

## Abbreviations

BC: Breast cancer
TCGA: The cancer Genome Atlas
GSEA: Gene set enrichment analysis
OS: Overall survival
MsigDB: Molecular Signatures Database
GLUT1: Glucose transporter type 1
GLUT3: Glucose transporter type 3
ER: Estrogen receptor
HER2: Human epidermal growth factor receptor 2
HPSE: Heparanase
MAP3K1: Mitogen-activated protein kinase kinase kinase 1
