# Supplementary figures and images for: Identification of novel cell glycolysis related gene signature predicting survival in patients with breast cancer
Source: Sci Rep. 2021 Feb 17;11:3986. doi: 10.1038/s41598-021-83628-9 (PMC7889867; doi:10.1038/s41598-021-83628-9)

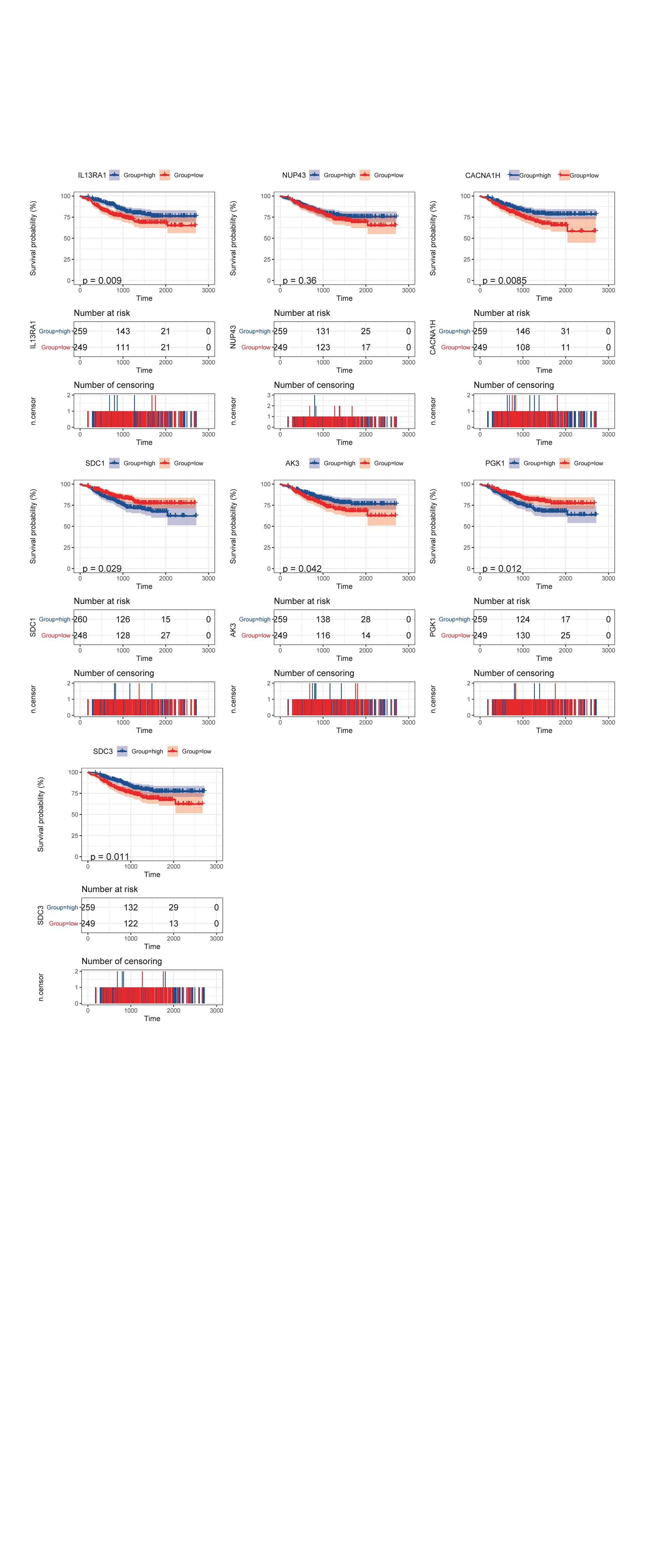

Supplement: Supplementary file 1 — Supplementary Figure S1. [file 41598_2021_83628_MOESM1_ESM.tif]
