# Supplementary material for: Identification of novel cell glycolysis related gene signature predicting survival in patients with breast cancer
Source: Sci Rep. 2021 Feb 17;11:3986. doi: 10.1038/s41598-021-83628-9 (PMC7889867; doi:10.1038/s41598-021-83628-9)
Supplement: Supplementary file 4 — Supplementary Legend. [file 41598_2021_83628_MOESM4_ESM.docx]

**Figure S1**.High and low expression of selected genes predict prognosis in BC patients
